# The First 1000 Days of Infant

**DOI:** 10.3390/healthcare10010106

**Published:** 2022-01-06

**Authors:** Juan Brines, Virginie Rigourd, Claude Billeaud

**Affiliations:** 1 Association Européenne de l’Enseignement de la Pédiatrie-European Association for Paediatrics Education (AEEP-EAPE) 49 Rue d’Alzon, 33000 Bordeaux, France; virginie.rigourd@nck.aphp.fr (V.R.); claude.billeaud@chu-bordeaux.fr (C.B.); 2 Department of Pediatric, Obstetrics and Gynecology, Faculty of Medicine, Valencia University, 46010 Valencia, Spain; 3 Neonatology and “Lactarium de l’ile de France” Necker-Enfants Malades Hospital, 75015 Paris, France; 4 Neonatology & Nutrition, CIC Pédiatrique 1401, INSERM, Hopital des Enfants, CHU Pellegrin Place Amelie Raba Leon, 33076 Bordeaux, France

The third edition of the Nursing and Pediatrics Congress was held in Paris from 16–19 June 2021, with the aim of contributing the experiences and reflections of relevant health professionals (pediatricians, pediatric surgeons, obstetricians, nurses, midwives, dieticians, and lactation consultants) to the knowledge of the most critical period of human life: its first 1000 days.

No other period of similar duration affects survival and quality of life so directly. During the thousand days that cover the entire intrauterine life and the first two years of extrauterine, the survival, growth and development of the human being is determined by a whole series of finely tuned genetic, environmental and social factors. 

Three stages of these first 1000 days of life of human beings incur particular vulnerabilities: the embryonic stage, the perinatal period, and that of lactation. The epigenetic and environmental aspects of the subject, as well as the impact of fetal programming on the growth and development of the preterm, were treated in detail by V. Rigourd from The Necker–Enfants Malades Hospital (Paris) and G. Gascoin from the hospital of Angers.

During the embryonic period (the first eight weeks of life), intense selective phenomena remove a high number of vital projects generated by fertilization.

It is estimated that only 50 to 60% of all conceptions exceed 20 weeks’ gestation. Approximately 70% of all pregnancy failures take place during the process of implantation of the zygote and therefore are not recognized as true pregnancies. The overall rate of gestational failure after implantation is 31% including clinically acknowledged miscarriages [1]. Maximum fertility, that is, the probability of conception during a menstrual cycle is approximately 30% [2]. Consequently, human reproduction shows considerable inefficiency [3] despite the indisputable growth of the world population observed since the middle of the last century.

The perinatal period (from the 28th week of gestation through to the 7th day after birth) follows the embryonic stage in terms of lethality [4]. The leading causes of perinatal mortality are prematurity, anoxia, and congenital malformations, to which are added, to a lesser extent, infections and metabolic disorders. Prematurity remains the main cause of infant mortality and morbidity [5]. Its frequency in developed countries ranges from 5 to 10% [6] and, in recent decades, its general trend has been to increase [7,8]. 

The main determinant of mortality and morbidity in premature infants is the poor adaptation to the extrauterine environment resulting from their immaturity which increases in line with the degree of prematurity. Immaturity, mainly through cellular hypoxia, is the main cause of mortality in large premature infants (extreme low weight, <1000 g), while congenital malformations assume greater prominence as the newborn (NB) approaches term birth [9].

The importance of cellular hypoxia secondary to respiratory disorders has decreased as a cause of neonatal mortality since the 1990s thanks to the therapeutic introduction of exogenous surfactant [10]. The therapeutically reduced mortality of premature infants due to surfactant deficiency has resulted in other conditions, formerly less frequent, assuming greater prominence. This is the case of necrotizing enterocolitis which, in the most severe cases, will require lifelong parenteral feeding with unsatisfactory results. Intestinal necrosis is frequently multiple and discontinuous, so respecting the intact intestinal segments as much as possible is required. A. López, C. Benlloch and J. Brines, from the Hospital Clínico de Valencia, offered their positive experience in the surgically conservative treatment of cases in which intestinal necrosis, although multiple, was discontinuous.

Advances in perinatal and neonatal care since the end of the 20th century have generally led to a marked reduction in perinatal, neonatal and infant mortality. The reduction in mortality has been more noticeable in large preterm infants [11,12,13,14].

The sequelae of the NBs assisted in hospitals, especially the neurological, sensory and cognitive ones, show figures that are in line with the mortality rate, so that the more preterm the delivery, the greater the number and severity of them.

Congenital deafness results in a serious sensory impairment in the normal socialization of the child. Cochlear implants and the optimization of audiological rehabilitation have constituted solid advances in the social integration of congenital deaf people, avoiding their evolution to deaf-muteness. J.M. Sequí and J. Brines, from the University of Valencia, updated delegates on the subject of diagnosis from their experience covering three decades.

The frequency of severe disabilities seems to have remained stable in recent years [12,15]. It should be noted, however, that the greatest successes in reducing perinatal mortality and morbidity have come from structural, diagnostic, and therapeutic advances in neonatal medicine. Among the former, it is worth emphasizing the role played by neonatal ICUs, whose increasing numbers during the 1970s promoted the dissemination of technical advances [16]. Surfactant therapy, the growth in lactation support and of human milk banks, transcutaneous O_2_ saturation measurement, routine ultrasound, pharmacological and surgical treatment of the ductus, as well as medical and surgical care for necrotizing enterocolitis, to name but a few, have been successfully introduced into these units. 

Progress in the prevention and care of preterm birth has been less marked, although the indisputable benefits derived from the careful selection of the mode of delivery, fetal monitoring, and maternal corticosteroids should not be forgotten.

For obvious reasons, nutrition has been a prominent aspect in this meeting that has emphasized the irreplaceable role of the mother in the health of the new being. The nutritional needs of the mother during pregnancy and lactation have been rigorously summarized in the presentation by C. Billeaud, from The University of Bordeaux and current President of the European Association for Paediatric Education. Following this line, C. Martinez -Costa and J. Estañ, from the University of Valencia, offered an update on the nutrition of the child in these first 1000 days and its projection onto the future adult.

Breastfeeding assumed great prominence in the meeting. Its evolutionary aspects were explained by J. Brines, University of Valencia, in the inaugural lecture. After an introduction provided by G. Weaver, co-founder of the Human Milk Foundation, an in-depth update on the importance of breastfeeding in infant nutrition was provided by M. Panard, Consultant of the International Board Lactation in Paris.

The associated factors to early breastfeeding attachment after C-section were presented by A.E. Njom Nlend from his broad and solid experience in the Maternity of Yaoundé (Cameroon). As expected, breastfeeding and human milk banking has not escaped the impact of the COVID-19 pandemic; a study of such influence has been the subject elaborated by N. Shenker from Imperial College of London. A main complication of breastfeeding is breast abscess; C. Bonnehon and M. Coicaud from the University of Bordeaux offer a detailed protocol for diagnosis and therapeutic follow-up based on their own extensive experience. Pain when breastfeeding is another difficulty that interferes with the normal nutrition of the infant; C. Elleau, from the University of Bordeaux, analyzed the subject and its relationship with osteopathy. 

M. Fayon from the University of Bordeaux contributed the most relevant characteristics of the development and pathology of the respiratory system during this study period. From the same university and in line with the rest of the conferences, M. Rebola addressed the important and complex subject of neurodevelopment and its intimate connection and temporal correspondence with the first stage of Piaget’s cognitive development in which the infant acquires knowledge and experience of himself and the environment through his sensory perceptions and motor activity.

Pre- and perinatal mortality, as we have advanced, is mainly due to pathological conditions related to prematurity, anoxia, and malformations. The burden of this type of disease decreases slowly over the first two years of life, with infections becoming more and more relevant. This aspect was updated and well summarized in the lecture by J. Sarlange of the University of Bordeaux.

As a complement to the central theme of the meeting, T. Szamosi of the Semmelweis University in Budapest addressed the role of free oxygen radicals as a pathogenic factor in some chronic conditions in young people.

We invite you to read the articles on the topic *The first 1000 days of the infant* that the referenced authors publish in this special issue of *Healthcare* MDPI.

## Data Availability

Not applicable.
